# Anatomical and visual outcomes of fovea-sparing internal limiting membrane peeling with or without inverted flap technique for myopic foveoschisis

**DOI:** 10.1186/s12886-022-02679-2

**Published:** 2022-11-18

**Authors:** Dezhi Zheng, Zijing Huang, Qin Zeng, Yifan Wang, Shirong Chen, Jingsheng Yi, Danqi Fang, Dingguo Huang, Weiqi Chen

**Affiliations:** Joint Shantou International Eye Center of Shantou University and the Chinese University of Hong Kong, 69# North Dongxia Road, Jinping District, Shantou, Guangdong 515041 P.R. China

**Keywords:** Myopic foveoschisis, Vitrectomy, Fovea-sparing, Internal limiting membrane

## Abstract

**Background:**

Vitrectomy and peeling of the internal limiting membrane (ILM) was an effective therapeutic approach for myopic foveoschisis with progressive visual loss. This study investigated the anatomical and visual outcomes of fovea-sparing ILM peeling with or without the inverted flap technique for patients with symptomatic myopic foveoschisis (MF).

**Methods:**

We retrospectively reviewed the clinical data of patients with MF. Vitrectomy with fovea-sparing ILM peeling and air tamponade was performed in all patients. The primary outcome measures included best-corrected visual acuity (BCVA), mean macular thickness (MMT), and central foveal thickness (CFT). Depending on whether an inverted ILM flap technique was utilized, further subgroup comparisons between the inverted flap group and the non-inverted flap group were conducted.

**Results:**

Twenty-six eyes of 22 patients were included. Fifteen eyes were underwent fovea-sparing ILM peeling without inverted ILM flap and 11 of the 26 eyes were treated with fovea-sparing ILM peeling and an inverted ILM flap technique. In the mean follow-up period of 10.74 ± 4.58 months, a significant improvement in BCVA was observed from 0.97 ± 0.45 logMAR to 0.58 ± 0.51 logMAR (*P* < 0.01), during which the BCVA of 20 eyes (76.92%) improved and remained stable in 5 eyes (19.23%). Moreover, a positive correlation was also found between the preoperative BCVA and the postoperative BCVA (*r* = 0.50, *P* = 0.01). At the last visit, the final MMT decreased from 492.69 ± 209.62 μm to 234.73 ± 86.09 μm, and the CFT reduced from 296.08 ± 209.22 μm to 138.31 ± 73.92 μm (all *P* < 0.01). A subgroup analysis found no significant differences in BCVA, MMT, or CFT between the inverted and non-inverted flap groups (all *P* > 0.05).

**Conclusion:**

Fovea-sparing ILM peeling with or without inverted flap technique resulted in favorable visual and anatomical outcomes for the treatment of MF. An important factor affecting the postoperative visual outcome was the preoperative visual acuity. Our study found no significant difference between the presence and absence of the inverted ILM flap.

**Supplementary Information:**

The online version contains supplementary material available at 10.1186/s12886-022-02679-2.

## Background

Approximately 9.80% of the world’s population was expected to have high myopia by 2050 [1]. A significant risk factor for visual loss was myopic foveoschisis (MF), which might develop in 9.00-34.40% of high myopia eyes with staphyloma [2, 3]. It was shown that the inward traction forces exerted by an epiretinal membrane and rigid ILM, and outward traction caused by excessive elongation of the eye and the posterior staphyloma, were both critical to the development of MF [4–6]. The natural course of MF progressed slowly, and visual acuity might remain stable for years [7]. However, serious complications can result in a significant loss of vision, such as foveal detachment (FD) and full-thickness macular hole (FTMH). Based on previous studies, 34.50–72.00% of patients with MF might develop FD [8, 9].

Pars plana vitrectomy and gas tamponade had received considerable discussion. Nevertheless, there was some controversy regarding the necessity and method of ILM peeling. It was shown that vitrectomy without ILM peeling was advantageous in treating MF eyes [10, 11]; however, it failed to completely release the macular interface traction [12, 13]. In contrast, complete ILM peeling followed by vitrectomy could release abnormal macular traction more effectively and was associated with a better anatomic outcome [14, 15]. Nevertheless, a postoperative macular hole formation rate of 8.00–18.00% has been reported following complete ILM peeling [16, 17]. In order to reduce the impact of surgical procedures on the macular structure, a foveal-sparing ILM peeling technique was proposed [18], which had better anatomic and visual outcomes for MF [19, 20].

The inverted ILM flap technique demonstrated an improvement in anatomic outcomes and functional outcomes for patients with myopic macular holes (MH) and high myopic macular hole induced retinal detachments [21, 22]. A flap of ILM covering the surface of MH stimulated the proliferation of glial cells and contributed to the restoration of foveal architecture as a scaffold [23]. Using the combined fovea-sparing ILM peeling and inverted flap technique, postoperative MH formation of MF was further prevented in a recent study [24].

Thus, this study aimed to investigate the anatomical and functional outcomes of fovea-sparing ILM peeling with or without the inverted ILM flap technique for the treatment of symptomatic MF.

## Methods

The research was conducted following the Declaration of Helsinki and approved by the Joint Shantou International Eye Center Ethics Committee. This retrospective and observational study reviewed the medical records of patients with MF who underwent vitrectomy with foveal-sparing ILM peeling from May 2018 to December 2021.

The inclusion criteria included subjects with MF over 18 years old, the presence of high myopia (spherical equivalent ≥ -6.0D or axial length ≥ 26.0 mm), and a progressive worsening of visual acuity because of increased MF severity or development of FD. The exclusion criteria were eyes with stable vision, full-thickness macular holes, choroidal neovascularization, previous vitreoretinal surgeries, or other fundus diseases impaired vision, such as diabetic retinopathy, retinal vein occlusion and glaucoma, etc. Additionally, patients who underwent silicone oil tamponade intraoperatively were also excluded. Finally, we enrolled twenty-six eyes of 22 patients with symptomatic MF.

Each patient received a comprehensive ophthalmologic examination, including measurements of the BCVA, refractive error and ocular biological parameters. The ocular axial lengths were measured with the intraocular lens Master Optical Biometer (IOL-Master 500, Carl Zeiss Meditec, Germany). The presence of posterior staphyloma was confirmed using B-scan ultrasonography. The morphological parameters of the retina were evaluated using optical coherence tomography (OCT, 3D OCT-2000, TOPCON, Japan). Based on the OCT scans, mean macular thickness (MMT), central foveal thickness (CFT) and the height of foveal detachment (HFD) were evaluated. The MMT was defined as the average thickness of the central retina with a diameter of 1 mm on the ETDRS grid map in the macular cube scan. Using a calliper tool on the OCT machine, the CFT and HFD were measured manually by the same technician. The CFT was defined as the distance from the inner to the outer surface of the neural retina at the fovea. The HFD was measured as the largest distance between the outer border of the neural retina and the inner border of the retinal pigment epithelium at the same site as for CFT. According to series OCT B-scans, a lamellar macular hole was diagnosed if there was foveal structural integrity damage accompanied with loss of inner or outer retina layer tissue, especially with the presence of ellipsoid line disruption [25]. A change in vision was defined as over one line change on Snellen vision chart on either direction (better or worse). The BCVA was converted to the logarithm of the minimum angle of resolution (logMAR) for statistical analysis.

**Fig. 1:**
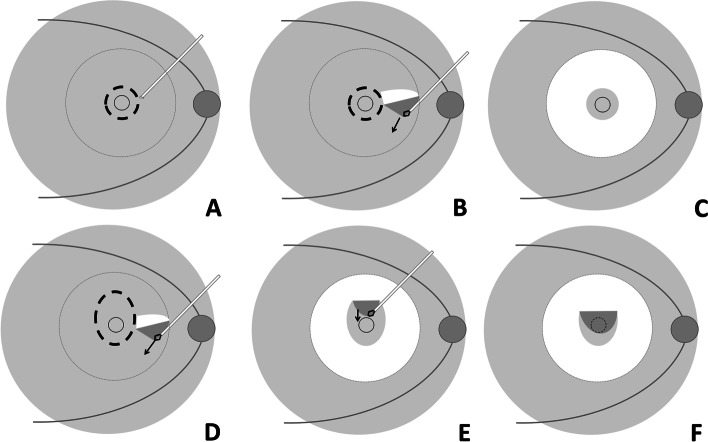
Schematic diagrams of fovea-sparing internal limiting membrane (ILM) peeling with and without inverted flap. **A** In the cases without inverted ILM flap, several sits of ILM tissue at the edge of the reserved area (bold dotted line) were ripped carefully at about one disc diameter away from the fovea, by the retrobulbar injection needle with a hook. **B** To precede along with the boundary of the reserved area. When the peeled ILM flap deviated from the boundary, to start ILM peeling from a new ripped site was available. **C** To get the ILM of posterior pole peeled off in the center preserved manner (dotted line circle). **D** In the cases with the inverted flap, an additional area about one disc diameter above the fovea was prepared when ripping the reserved boundary (bold dotted line). **E** After removing the ILM around the reservation area, the above additional ILM was peeled and inverted towards to the foveal zone. **F** The ILM forceps covered the prepared ILM flap onto the fovea area

The surgical approach was a standard 23 gauge 3-port pars plana vitrectomy performed by the same experienced vitreous surgeon. Depending on the status of the lens, phacoemulsification and implantation of a mono-focal intraocular lens were applied. After removing the vitreous, the posterior boundary membrane of vitreous and epimacular membrane were entirely removed if it existed, and then indocyanine green (2 mg/mL for about 10 s) was employed to visualize the ILM. With the help of microscope, a slight hook was artificially made on the tip of a disposable retrobulbar injection needle (23 gauge, 38 mm; Kindly Enterprise Development Group Co., Ltd., Shanghai, China) by pressing it slightly against the side wall of forceps. The angle of the hook was about 90º. The edge of the foveal ILM was ripped discontinuously in one optic disc diameter by the hook. After advancing the edge of the target region with the hook, the posterior pole area ILM was removed with the ILM forceps in a centrally preserved manner (Additional file 1). For the inverted ILM procedure, extra superior ILM tissue about one optic disc diameter was reserved and inverted onto the fovea before the fluid-air exchange (Fig. 1). Retinal photocoagulation was performed in the cases with retina tears or lattice degenerations. Tamponade with filtered air was performed at the end of the procedure, and the patients were instructed to maintain a prone position for 1 week.

In this study, the primary outcome measurements were the BCVA, MMT and CFT. Quantitative data were presented as means ± standard deviations. The BCVA, MMT, CFT, and HFD were compared between baseline and the last follow-up using a Wilcoxon signed rank test. According to the normality test (Shapiro–Wilk test) and homogeneity test (Levene test), student t-test or Mann-Whitney U test was used to compare the differences of preoperative and postoperative parameters between the inverted and non-inverted flaps groups. Using Spearman correlation coefficient, an estimation of the correlation between preoperative parameters and postoperative BCVA was performed. The statistical analysis was performed using the SPSS for Windows version 11.5 (SPSS Inc, Chicago, Illinois, USA). *P* values of less than 0.05 were considered statistically significant.

## Results

Twenty-six eyes of 22 patients (17 females) were enrolled and 25 eyes underwent phacoemulsification, and intraocular lens implantation. Table 1 summarized the demographic data of the subjects. Nineteen eyes (73.08%) had lamellar macular holes, and 9 eyes (34.62%) had foveal detachment. Moreover, posterior scleral staphyloma was present in all eyes. There was a mean follow-up period of 10.74 ± 4.58 months. The eyes were further divided into the inverted flap group (*N* = 11) and the non-inverted flap group (*N* = 15) based on whether or not an inverted ILM flap technique was used.

**Table 1:** Demographic data and follow-up after fovea-sparing ILM peeling

	Preoperative (*N* = 26)	Last follow-up (*N* = 26)	*P*
**Age**/years mean ± SD(range)	56.81 ± 9.33 (41.00 to 80.00)	/	/
**Axial length**/mm mean ± SD(range)	29.53 ± 1.30 (26.97 to 31.94)	/	/
**Mean spherical equivalen**t/D mean ± SD(range)	-13.90 ± 4.28 (-6.50 to -22.25)	/	/
**BCVA**/logMAR mean ± SD(range)	0.97 ± 0.45 (0.30 to 1.85)	0.58 ± 0.51 (0 to 1.85)	<0.01^*^
**MMT**/µm mean ± SD(range)	492.69 ± 209.62 (169.00 to 1241.00)	234.73 ± 86.09 (69.00 to 407.00)	<0.01^*^
**CFT**/µm mean ± SD(range)	296.08 ± 209.22 (101.00 to 840.00)	138.31 ± 73.92 (24.00 to 353.00 )	<0.01^*^
**HFD**/µm mean ± SD(range)	326.11 ± 304.52 (45.00 to 1030.00 )	60.22 ± 86.65 (0 to 252.00)	<0.01^*^

Wilcoxon signed rank test

*ILM* Internal limiting membrane, *BCVA* Best corrected visual acuity, *logMAR* Logarithm of the minimum angle of resolution, *LMH* Lamellar macular hole, *MMT* Mean macular thickness, *CFT* Central foveal thickness, *HFD* Height of foveal detachment

The BCVA was significantly improved at the final visit from 0.97 ± 0.45 logMAR to 0.58 ± 0.51 logMAR (*P* < 0.01). The BCVA improved in 20 of the 26 eyes (76.92%) and remained stable in 5 eyes (19.23%). Table 1 showed the significant improvement in MMT, CFT and HFD. The FD was restored entirely in four of the nine eyes (44.44%). The preoperative BCVA showed a moderate correlation with the BCVA at the last visit (*r* = 0.50, *P* = 0.01). In contrast, the axial length, preoperative MMT and CFT showed no correlation with the postoperative BCVA (r_AL_= 0.03, r_MMT_ = –0.09, r_CFT_= 0.05, all *P* > 0.05).

**Fig. 2:**
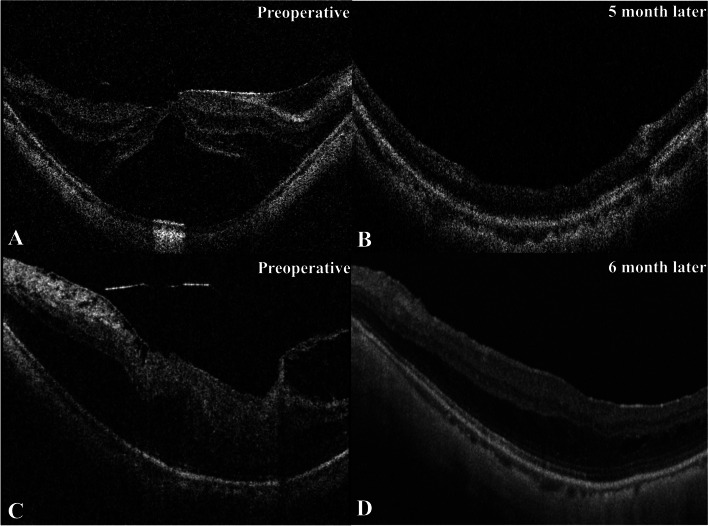
OCT images of the patients who underwent fovea-sparing internal limiting membrane (ILM) peeling with and without inverted ILM flap. **A** Preoperative OCT scan indicated severe foveal detachment (FD) in a fifty years-old female, who had received fovea-sparing ILM peeling with an inverted ILM flap. **B** Five months after the surgery, the FD got complete recovery and the best-corrected visual acuity (BCVA) improved from 1.30 logMAR to 0.40 logMAR. **C** Preoperative OCT scan showed foveoschisis and inner retinal structure disorder in a fifty -two-year-old female, who had received fovea-sparing ILM peeling without inverted ILM flap. **D** Six months later, the degree of foveoschisis reduced and the morphology of the retina got improved, with the BCVA improved from 1.00 logMAR to 0.05 logMAR

Considerable improvements were observed in the morphological structure both in the inverted flap group and the non-inverted flap group (Fig. 2). The subgroup analysis did not reveal significant differences between the inverted and non-inverted flap groups on the postoperative BCVA, MMT, or CFT (Table 2).

**Table 2:** Comparation of visual acuity and anatomic outcomes between inverted flap group and non-inverted flap group

	Inverted flap group (*N* = 11)	Non-inverted flap group (*N* = 15)	*P*
**Age**/years mean ± SD (range)	54.64 ± 7.21 (41.00 to 66.00)	58.40 ± 10.52 (43.00 to 80.00)	0.32
**No. of FD**/ n (%)	5 (45.45%)	4 (26.67%)	0.42^&^
**No. of LMH**/ n (%)	9 (81.82%)	10 (66.67%)	0.66^&^
**Axial length**/mm mean ± SD(range)	29.51 ± 1.32 (26.97 to 31.65)	29.55 ± 1.33 (27.63 to 31.94)	0.93
**Follow-up period**/ monthsmean ± SD (range)	9.20 ± 3.31 (4.30 to 16.60)	11.86 ± 5.14 (3.13 to 18.33)	0.19^#^
**Preoperative**			
** BCVA**/logMARmean ± SD(range)	1.02 ± 0.38 (0.52 to 1.85)	0.93 ± 0.51 (0.30 to 1.85)	0.66
** MMT**/µm mean ± SD(range)	438.36 ± 137.51 (169.00 to 609.00)	532.53 ± 246.85 (261.00 to 1241.00)	0.07^#^
** CFT**/µm mean ± SD(range)	237.91 ± 215.52 (101.00 to 840.00)	338.73 ± 200.96 (123.00 to 766.00)	0.35^#^
**Last follow-up**			
** BCVA**/logMAR mean ± SD(range)	0.66 ± 0.52 (0.10 to 1.85)	0.55 ± 0.49 (0.10 to 1.85)	0.50^#^
** MMT** /µm mean ± SD(range)	215.36 ± 100.77 (69.00 to 407.00)	249.36 ± 83.25 (143.00 to 376.00)	0.37
** CFT**/µmmean ± SD(range)	138.46 ± 89.20 (24.00 to 353.00)	138.20 ± 63.81 (40.00 to 298.00)	0.99

*P*^&^, Fishers exact test; *P*, Student-t test; *P*^#^, Mann-Whitney U test

*BCVA* Best corrected visual acuity, *logMAR* Logarithm of the minimum angle of resolution, *MMT* Mean macular thickness, *CFT* Central foveal thickness, *FD* Foveal detachment, *LMH* Lamellar macular hole

**Fig. 3:**
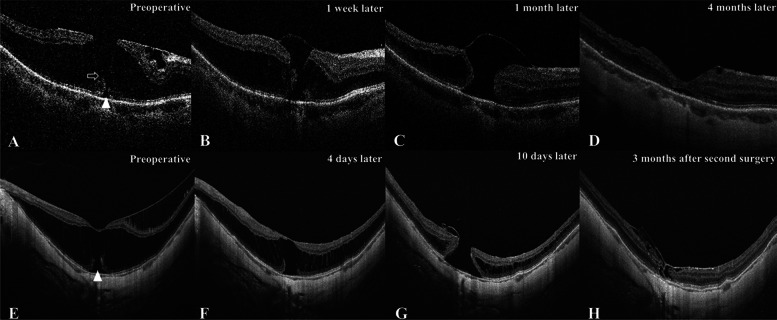
OCT images of two patients who developed full-thickness macular hole postoperatively (**A-D**) Sixty-six years-old female with an axial length of 26.97 mm had received fovea-sparing internal limiting membrane (ILM) peeling with inverted ILM flap. The preoperative best-corrected visual acuity (BCVA) was 0.52 logMAR and stable at the last visit. (Panel **A**) The preoperative scan indicated obvious epimacular membrane traction, ellipsoid line disruption (white triangle) and thin bridge of tissue in the inner retina layer (white arrow). (Panel **B**) A full-thickness macular hole (MH) was discovered one week after surgery with a diameter of 151 μm. (Panel **C**) The MH was enlarged, and the ILM flap was still covering the surface of the MH one month after surgery. (Panel **D**) The MH closed automatically about four months after the surgery without further surgical treatment. (**E-H**) Forty-four-year-old female with an axial length of 27.70 mm had received fovea-sparing ILM peeling without inverted ILM flap. The postoperative BCVA decreased from 0.60 logMAR to 1.85 logMAR. (Panel **E**) The preoperative scan showed a serve ellipsoid line disruption (white triangle). (Panel **F**) The degree of foveoschisis was relieved at four days after surgery. (Panel **G**) A full-thickness MH with diameter of 425 μm was discovered ten days after surgery. (Panel **H**) The MH was closed with ILM tissue insertion three months after the second surgery, which including ILM inserted, inverted ILM flap and air tamponade three months after the primary surgery

In both the inverted and non-inverted flap groups, one eye developed delayed onset FTMH after the operation. Another surgical procedure was performed three months later in the eye without an inverted ILM flap, and the MH was closed following the last visit. Meanwhile, the MH closed automatically four months after the surgery in the eye with an inverted ILM flap (Fig. 3).

## Discussion

Our study showed that phacovitrectomy and fovea-sparing ILM peeling with or without inverted ILM flap could significantly improve the anatomical and functional outcomes in eyes with MF. The preoperative visual acuity played an important role in the visual outcome.

Pars plana vitrectomy combined with fovea-sparing ILM peeling could reduced the risk of FTMH formation in vitreomacular interface diseases [26]. ILM peeling with foveal retention avoided damaging central Müller cells, which were connected tightly to the photoreceptor cells, therefore reducing the risk of postoperative macular alterations [27]. The inverted ILM flap technique was initially used to treat MHs, and proved to be effective in achieving higher closure rates of the large macular holes [28] and improving anatomical and functional outcomes in myopic macular holes and high myopic macular holes accompanied by retinal detachment [21, 22]. A recent study indicated combining fovea-sparing ILM peeling and inverted ILM flap technique further reinforced the foveal structure and decreased the risk of FTMH formation in MF patients [29].

The severity of foveoschisis in all patients was reduced in our study.The BCVA improved from 0.97 ± 0.45 logMAR to 0.58 ± 0.51 logMAR at the final visit, and 20 out of 26 eyes achieved visual acuity improvement. Additionally, it was shown that the preoperative BCVA was an essential predictor of the surgical outcome. This result was in agreement with the previously published findings [30, 31]. All eyes with FD were relieved of the severity, but only four eyes (44.44%) acquired complete recovery. This might be relevant to the relatively short follow-up period in our study. There were no significant differences in the postoperative visual acuity and macular morphologic parameters between the groups with and without the inverted ILM flap technique. A small sample size might affect the statistical analysis. However, the results indicated that preoperative visual acuity could be the essential factors to the surgical outcomes for MF.

MF was mainly associated with one or more macular abnormalities, including macular traction, foveal detachment and lamellar macular hole [8]. Fovea-sparing ILM peeling reduced the risk of structural damage to the macular by avoiding mechanical traction on the foveal region. Vitrectomy combined with fovea-sparing ILM peeling could improve postoperative visual acuity and central retinal sensitivity [32]. The foveal sparing ILM peeling and complete ILM peeling in MF were compared by Shimada and Iwasaki [18, 20]. Their studies showed that the BCVA was better and MH formation incidence was lower in the fovea-sparing ILM peeling group. In this study, we started the foveal preserved ILM peeling by discontinuously ripping the border of target region using a retrobulbar injection needle. It helped to determine the area of reserved ILM more easily, and prevent from peeling excessively during the operation. The ILM flap was reserved in one optic disc diameter to cover the fovea appropriately, and to avoided shrinkage because of excessive reserved area. The pre-existing cataract and increase in the cataract after surgery could affect visual prognosis. To achieve better visual prognosis, the combined phacoemulsification with vitrectomy was a preferable strategy for those patients with MF and cataract.

Lin et al. [29] reported significant anatomical and functional improvement using combined fovea-sparing ILM peeling with ILM flap for myopic traction maculopathy. The incidence of postoperative FTMH formation was obviously lower in the combined techniques group. However, the difference in the improvement of BCVA and macular thickness was insignificant between the fovea-sparing ILM peeling group and combined techniques group. Fovea-sparing ILM peeling did not guarantee to complete the absence of MH formation. Previous studies reported an incidence of FTMH formation after fovea-sparing ILM peeling ranging from 5.60 to 9.70%. Additionally, the preoperative presence of a lamellar macular hole was associated with a significantly increased risk of developing postoperative FTMH [29, 33]. We observed two cases developing postoperative FTMH in our study, which occurred approximately one week after the operation. Preoperative OCT scans revealed outer retina layer disruption in the two patients (Fig. 3). The vision got worse in the case who underwent secondary surgery, though the MH was closed at the final follow-up. The choroidal atrophy, distortion of macular architecture and loss of tissue, and potential photic toxicity of re-staining of ILM during the second operation were potential risk factors for the diminution of vision. The formation of FTMH occurred with the restoration of MF, and the intraoperatively direct damage on the fovea should not be blamed. One possible explanation was that the inner retina became relatively deficient because of the scleral staphyloma, and the tension on the tangent increased during the attachment of the inner retina. A severe staphyloma contour irregularity might enhance this procedure. The displacement of inner retina reattaching towards outer layer of retina became uneven at the irregular staphylomatous area, so the interfacial tension might increase at the site where scleral curvature changed greatly.

There were several limitations in our study. Firstly, it was not a prospective cohort study, and there was no random sampling. Additionally, the sample size was relatively small due to the low morbidity of symptomatic MF and the lack of surgical willingness of the patients.

In conclusion, our study indicated that fovea-sparing ILM peeling with or without the inverted ILM flap technique was appropriate for treating MF. A major factor affecting the visual prognosis was the preoperative visual acuity. Further prospective controlled trials were necessary to evaluate the advantage of the inverted ILM flap technique for myopic foveoschisis.

## Supplementary Information

Additional file 1: **Video 1. **Procedure for fovea-sparing internal limiting membrane (ILM) peeling with inverted flap. After indocyanine green staining, several sits of the ILM tissue at the edge of the reserved area were ripped carefully at about one disc diameter away from the fovea by a retrobulbar injection needle with tiny hook at the tip. Additional area about one disc diameter above the fovea was prepared when ripping the reserved boundary. To Peel the ILM tissue of posterior pole along with the boundary of the reserved area. To start ILM peeling from a new ripped site was available if the peeled ILM flap deviated from the boundary. Following the removal of the ILM around the reservation area, the above additional ILM was peeled and inverted onto the foveal region.

## Data Availability

All data supporting these findings are contained within this manuscript.
